# Identification and validation of an immune-related gene pairs signature for three urologic cancers

**DOI:** 10.18632/aging.203886

**Published:** 2022-02-10

**Authors:** Biao Xie, Kangjie Li, Hong Zhang, Guichuan Lai, Dapeng Li, Xiaoni Zhong

**Affiliations:** 1 Department of Biostatistics, School of Public Health and Management, Chongqing Medical University, Chongqing, China; 2 Institute of Hepatology, National Clinical Research Center for Infectious Disease, Shenzhen Third People’s Hospital, Shenzhen, China

**Keywords:** urologic cancer, bladder cancer, prostate cancer, kidney cancer, immune-related gene pairs

## Abstract

Reliable biomarkers are needed to recognize urologic cancer patients at high risk for recurrence. In this study, we built a novel immune-related gene pairs signature to simultaneously predict recurrence for three urologic cancers. We gathered 14 publicly available gene expression profiles including bladder, prostate and kidney cancer. A total of 2,700 samples were classified into the training set (n = 1,622) and validation set (n = 1,078). The 25 immune-related gene pairs signature consisting of 41 unique genes was developed by the least absolute shrinkage and selection operator regression analysis and Cox regression model. The signature stratified patients into high- and low-risk groups with significantly different relapse-free survival in the meta-training set and its subpopulations, and was an independent prognostic factor of urologic cancers. This signature showed a robust ability in the meta-validation and multiple independent validation cohorts. Immune and inflammatory response, chemotaxis and cytokine activity were enriched with genes relevant to the signature. A significantly higher infiltration level of M1 macrophages was found in the high-risk group versus the low-risk group. In conclusion, our signature is a promising prognostic biomarker for predicting relapse-free survival in patients with urologic cancer.

## INTRODUCTION

Bladder cancer, prostate cancer and kidney cancer are the main tumors in the urinary system, and nearly 2.4 million new cases are diagnosed each year [1]. The advance of targeted therapy and neoadjuvant therapy has prolonged the survival of patients [2–4]. However, numerous patients suffer relapses even after complete surgical resection [5–7], and their prognoses are still not optimistic. A reliable prognostic biomarker which could identify patients with a higher risk for relapse and select patients who have response to therapies would be valuable for management of urologic cancers.

Gene-expression signatures have been identified for survival stratification of bladder cancer [8, 9], prostate cancer [10, 11] and kidney cancer [12, 13]. However, most biomarkers have not been translated to clinical practice due to over-fitting of training datasets or lack of sufficient validation [14]. A chance to develop more reliable prognostic biomarkers has been brought by sufficient large-scale public gene expression datasets [15, 16]. However, it is a challenge to integrate data derived from different platforms. The traditional method has made it difficult to normalize different datasets, given technical biases and biological heterogeneity of multiple platforms [17, 18]. New methods based on the relative ranking of gene expression levels have been used to eliminate the requirement for data preprocessing, and have attained robust results in many applications [19–21].

Increasing evidence has indicated that the tumor immune microenvironment is correlated with the formation and progression of the three main urologic tumors [22–24]. The immune checkpoint molecules, such as programmed cell death 1 (PD-1), PD-1 ligand 1 (PD-L1) and cytotoxic T-lymphocyte associated antigen 4 (CTLA-4), have demonstrated a remarkable, durable response in bladder cancer [25, 26], prostate cancer [27, 28] and kidney cancer [29, 30]. The biomarkers related with the tumor immune microenvironment may thus have potential as prognostic markers of urologic cancers.

As is well-known, bladder cancer, prostate cancer and kidney cancer are closely related anatomically and result from similar insults that promote tumor formation [31–33]. Therefore, we have developed in this study a signature based on immune-related gene pairs (IRGPs) to simultaneously predict the recurrence of bladder cancer, prostate cancer and kidney cancer.

## MATERIALS AND METHODS

### Study design and datasets

We comprehensively analyzed 14 gene expression profiles in three urologic tumors of bladder, prostate and kidney cancer, including seven microarray datasets and seven RNA-Seq datasets (Supplementary Figure 1). The accession numbers, platforms and samples sizes of these gene expression profiles are shown in Supplementary Table 1. RNA-Seq data were downloaded from UCSC Xena (http://xena.ucsc.eduaccessed on January 2021) and the International Cancer Genome Consortium (ICGC) (https://dcc.icgc.org/projects). Microarray data were downloaded from the Gene Expression Omnibus (GEO) (http://www.ncbi.nlm.nih.gov/geo). Cohorts from The Cancer Genome Atlas (TCGA) were used as the training set, and other datasets were used as the validation set. Only patients with complete survival information were included. We also excluded patients who had received radiation therapy, neoadjuvant therapy and targeted molecular therapy in all independent training cohorts. In total, 2,700 cases were included in our study. Our project was approved by Chongqing Medical University’s Ethical Review Committee.

### Gene expression data processing

The publicly available datasets from GEO were firstly normalized using the normalizeBetweenArrays function as implemented in the ‘limma’ package, and then were further log-transformed. Normalization methods were not used in TCGA and ICGC cohorts.

### Identification of specific IRGPs for prognosis prediction

We downloaded immune-related genes (IRGs) from the ImmPort database (https://immport.niaid.nih.gov) accessed on 3/3/2021. 2,483 unique IRGs, constituting 17 categories, including cytokines, cytokine receptors, antigen processing, presentation pathways, interleukins, natural killer cell cytotoxicity, TGFb and TNF family members. Only IRGs measured by all platforms with a median absolute deviation > 0.5 in all independent training sets were chosen. The score for each IRGP was generated by pairwise comparisons of the gene expression level in a certain sample of profiles. The IRGPs score was defined as 1 if expression level of IRG _1_ was larger than IRG _2_; otherwise, the IRGPs score was set as 0 [34]. After removing IRGPs with constant values in any individual dataset, the remaining IRGPs were further analyzed.

### Construction of the immune-related gene pairs index (IRGPI) for prognosis prediction

Prognostic IRGPs were selected based on the following steps. Firstly, the predictive ability of each IRGP predicting patients’ relapse-free survival (RFS) was evaluated by using the Cox regression model in the meta-training dataset, and the IRGPs with a p-value < 0.05 were selected as initial candidate markers. Secondly, the least absolute shrinkage and selection operator (LASSO) analysis was utilized to further filter out some less informative IRGPs. The tuning parameter was determined by the expected generalization error estimated from 10-fold cross-validation. To improve robustness, we randomly split the full meta-training dataset into new training and testing datasets with a 2:1 ratio, and repeated the random split scheme 30 times to stabilize the IRGPs selection procedure. The LASSO model was then applied to the 30 training sets, and those IRGPs with a frequency > 15 were selected. Finally, the multivariate Cox regression model was used to build the IRGPs-based prediction model and generate the IRGPI for all samples. The patients were classified into low and high immune risk groups using the median IRGPI value.

### Evaluation and validation of the IRGPI

The prognostic value of the IRGPI was evaluated in the meta-training and independent training sets, and was further verified in the meta-validation and multiple independent validation sets. The log-rank test and time-dependent receiver operating characteristic (ROC) curves were adopted to assess the prognostic accuracy of the IRGPI. We combined IRGPI with clinical factors of age, gender and tumor stage in multivariate Cox analyses. Age (>60) was transformed into 1, age (<60) was transformed into 0. Stage III and IV were transformed into 1, stage I and II were transformed into 0.

### Profiling of infiltrating immune cells

CIBERSORT characterizes immune cell composition by using bulk-tumor gene expression profiles [35]. It inferred the relative proportions of 22 types of infiltrating immune cells based on the reference gene expression values (LM22) [35]. In this study, the proportions of 22 infiltrating immune cells were determined by using the R package ‘CIBERSORT’. The perm was set at 1,000, and cases with a CIBERSORT output p-value < 0.05 were selected for further analysis. The Wilcoxon rank sum test was utilized to compare differences in immune cell subtypes between the high and low IRGPI groups.

### Gene ontology (GO) analysis

The R package ‘clusterProfiler’ was utilized to conduct GO enrichment analysis of the genes related to the IRGPI in the meta-training cohort. The Benjamini-Hochberg-adjusted p-value < 0.05 (false discovery rate, FDR) was used as the threshold to determine significance.

### Construction and evaluation of the nomogram

A nomogram was constructed to establish a quantitative approach for RFS prediction in the meta-training cohort based on the IRGPI and clinical factors, which was further verified in the meta-validation cohorts. A point was calculated for each factor, and the total points of all factors were then obtained for the estimation of RFS rates at 1, 3, 5, and 10 years. The calibration plots were then drawn to evaluate the reliability of the nomogram.

### Statistical analysis

The R package ‘survival’, ‘glmnet’, ‘survminer’, ‘timeROC’, ‘rms’, ‘CIBERSORT’ and ‘clusterProfiler’ were used to construct the Cox regression model, LASSO model, Kaplan-Meier curve, time-dependent ROC curve, nomogram, immune cell composition computation and GO analysis. The association of IRGPI score with RFS was analyzed by log-rank test. The Cox regression model was adopted to perform multivariate analysis of the association of IRGPI with RFS. A two-sided p-value < 0.05 was considered statistically significant for all tests. All statistical analyses were conducted using R (version 4.0.2).

## RESULTS

### Establishing and evaluating the IRGPI

**Figure 1:**
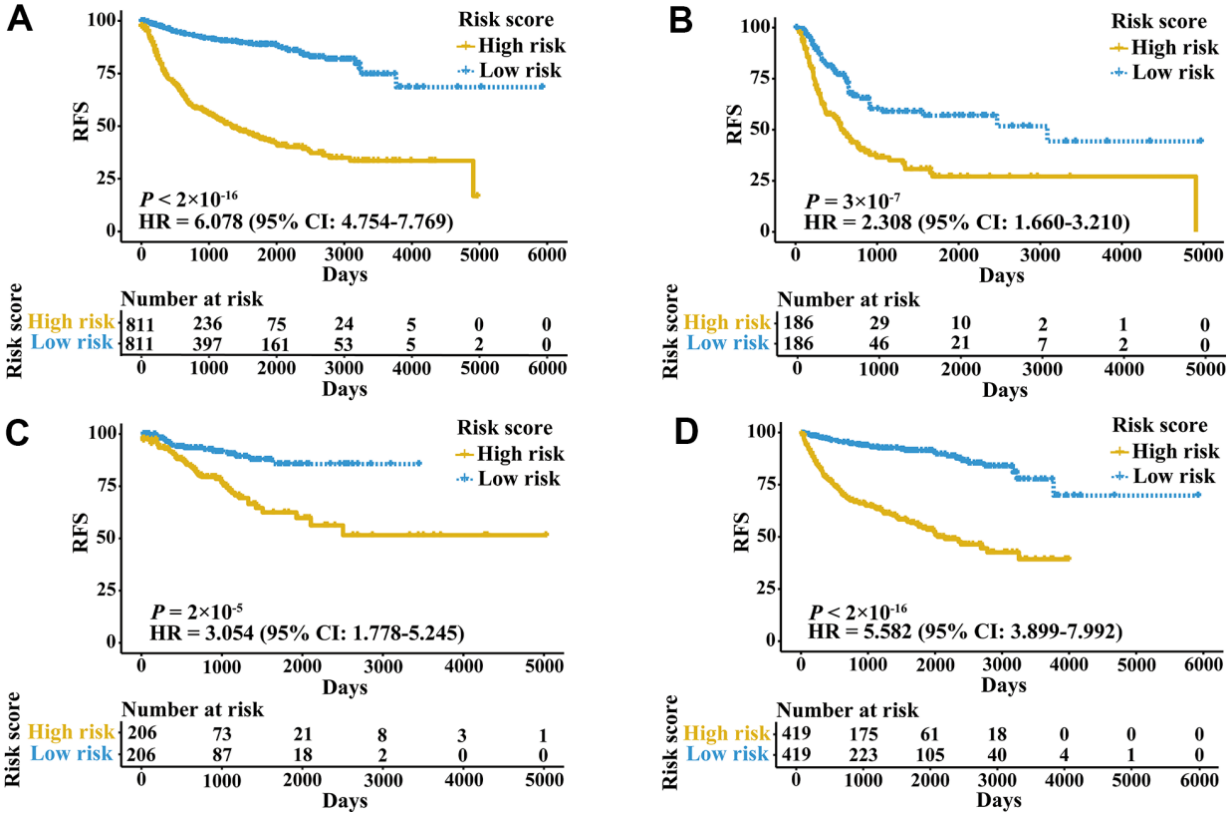
**Kaplan-Meier curves of patients in training cohorts stratified by the IRGPI.** (**A**) RFS among patients in the meta-training cohort. (**B**) RFS among patients in the training cohort of bladder cancer. (**C**) RFS among patients in the training cohort of prostate cancer. (**D**) RFS among patients in the training cohort of kidney cancer. HRs and 95% CIs are shown for high vs low immune risk. *P* values comparing risk groups were calculated by the log-rank test.

**Figure 2:**
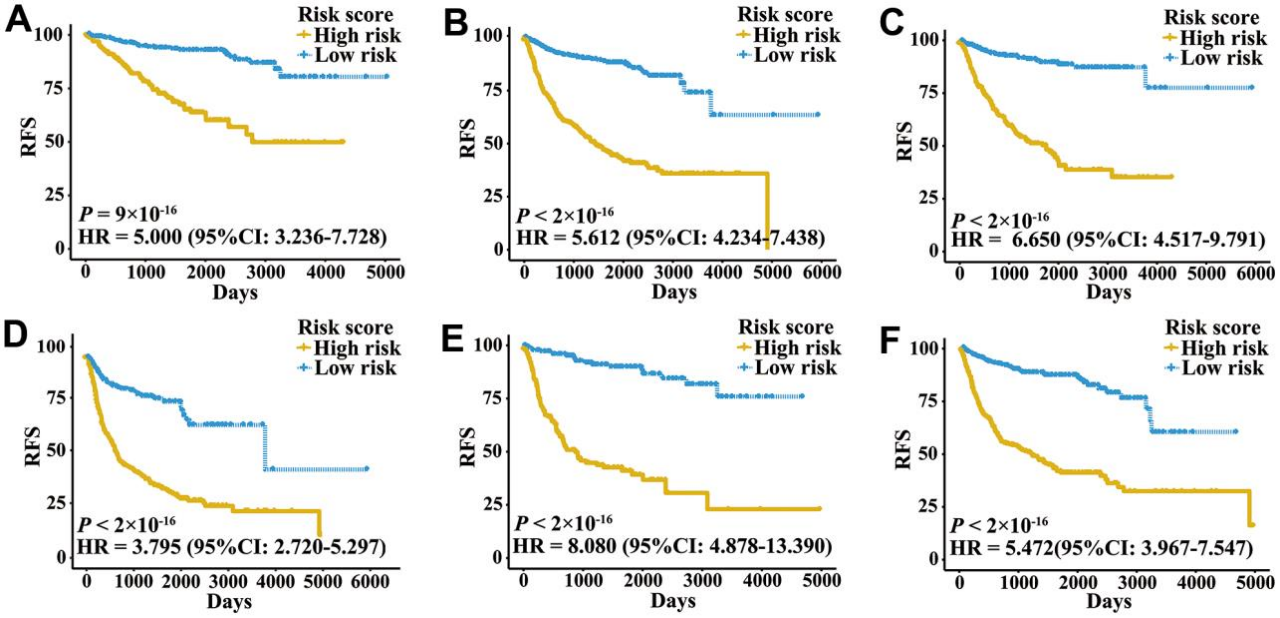
**Kaplan-Meier curves of patients with different clinical factors in the meta-training cohort.** (**A**) RFS among patients with early-stage disease. (**B**) RFS among male patients. (**C**) RFS among patients younger than 60 years. (**D**) RFS among patients with late-stage disease. (**E**) RFS among female patients. (**F**) RFS among patients older than 60 years.

A total of 2,700 patients with three urologic cancers, including 835 bladder cancer, 888 prostate cancer and 977 kidney cancer patients, were included in this study (Supplementary Table 1). As shown in Supplementary Figure 1, 1,622 patients in TCGA cohorts were used as the meta-training dataset. Another 1,078 patients from seven GEO datasets and two ICGC datasets constituted the meta-validation dataset. Among 2,483 IRGs from the ImmPort database, 606 IRGs were measured on all platforms and met the criteria (MAD > 0.5) on all independent training sets. Based on 606 IRGs, 183,315 IRGPs were constructed. After removing those not shared among all datasets or with constant ordering in any data set, 18,041 IRGPs were left and selected for further analysis. First, 10,943 IRGPs were filtered out by using the univariate Cox regression model in the meta-training data set. To further screen candidate IRGPs, LASSO was conducted 30 times to select those which appeared more than 15 times out of 30 analyses. As a result, 25 IRGPs and 41 unique IRGs were selected (Supplementary Table 2). The multivariate Cox regression model was then used to obtain the IRGPs-based prediction model and generate IRGPI scores for all samples. The patients in the meta-training cohort were classified into low and high immune risk groups by using the median IRGPI (-1.216973). There were significantly different prognoses in terms of RFS between low- and high-risk groups in the meta-training cohort (Figure 1A, hazard ratio [HR] 6.078, 95% confidence interval [CI] 4.754-7.769; *P* < 2×10^-16^). The IRGPI could also divided patients into subgroups with significantly different RFS in the training cohort of bladder cancer (Figure 1B, HR 2.308, 95% CI 1.660-3.210; *P* = 3×10^-7^), prostate cancer (Figure 1C, HR 3.054, 95% CI 1.778-5.245; *P* = 2×10^-5^) and kidney cancer (Figure 1D, HR 5.582, 95% CI 3.899-7.992; *P* < 2×10^-16^). The time-dependent ROC curves were used to evaluate the ability of the IRGPI to predict prognosis. The area under the curve (AUC) values in predicting 1-, 3-, 5- and 10-year RFS of patients were 0.816, 0.818, 0.828, and 0.763, respectively, in the meta-training cohort (Supplementary Figure 2A). When patients were stratified by different tumor stages, genders and age groups, low and high IRGPI groups remained significantly different for RFS, and a higher IRGPI score was associated with significantly worse prognosis (Figure 2). The patients with high IRGPI and advanced tumor stage had the highest RFS among all patients in the meta-training cohort (Supplementary Figure 2B). Multivariate analyses suggested that the IRGPI was an independent prognostic factor (HR 4.22, 95% CI 3.23-5.52; *P* < 2×10^-16^) after adjusting for age and stage (Supplementary Table 3).

### Validation of the IRGPI

**Figure 3:**
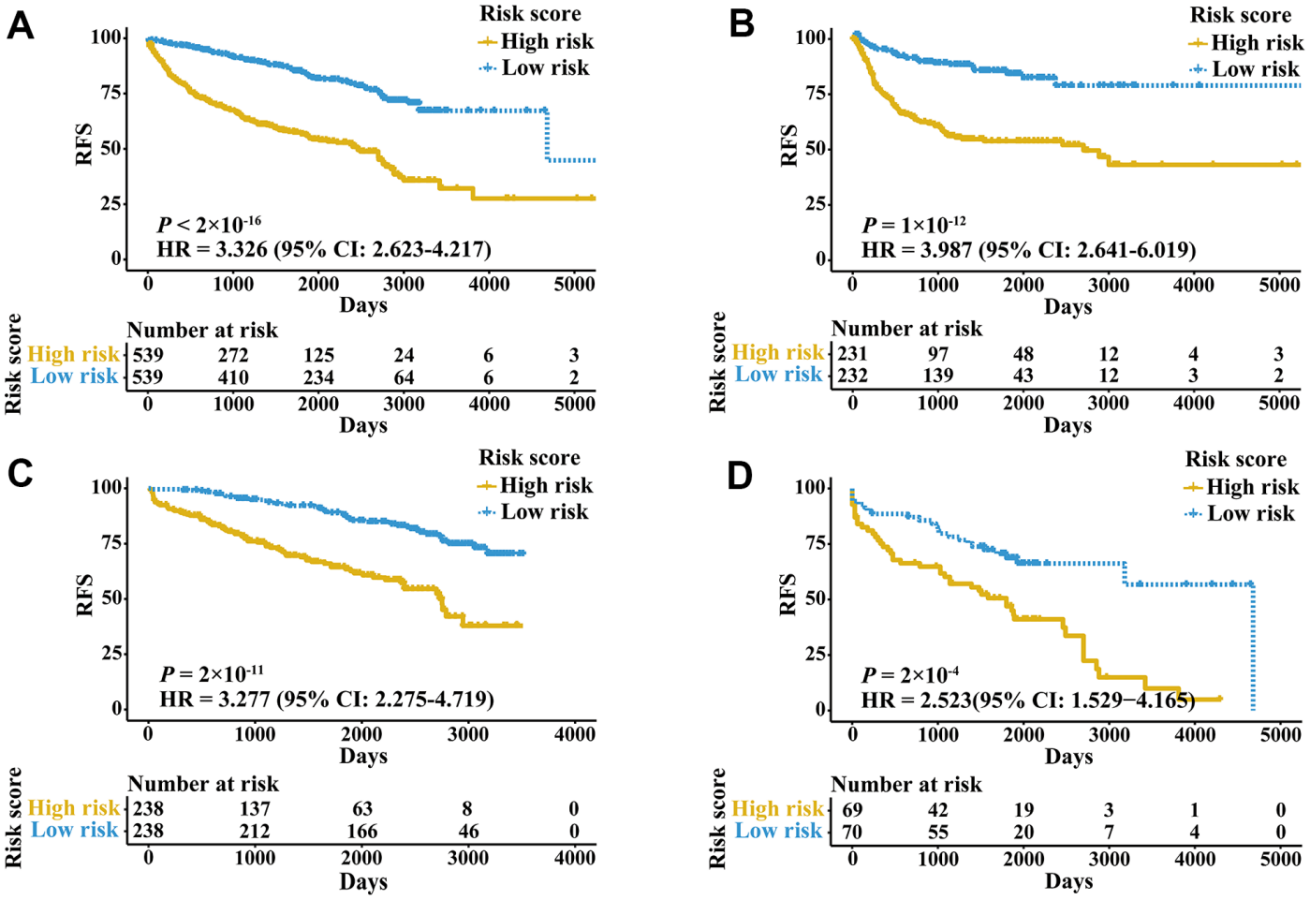
**Kaplan-Meier curves of patients in validation cohorts stratified by the IRGPI.** (**A**) RFS among patients in the meta-validation cohort. (**B**) RFS among patients in the validation cohort of bladder cancer. (**C**) RFS among patients in the validation cohort of prostate cancer. (**D**) RFS among patients in the validation cohort of kidney cancer.

**Figure 4:**
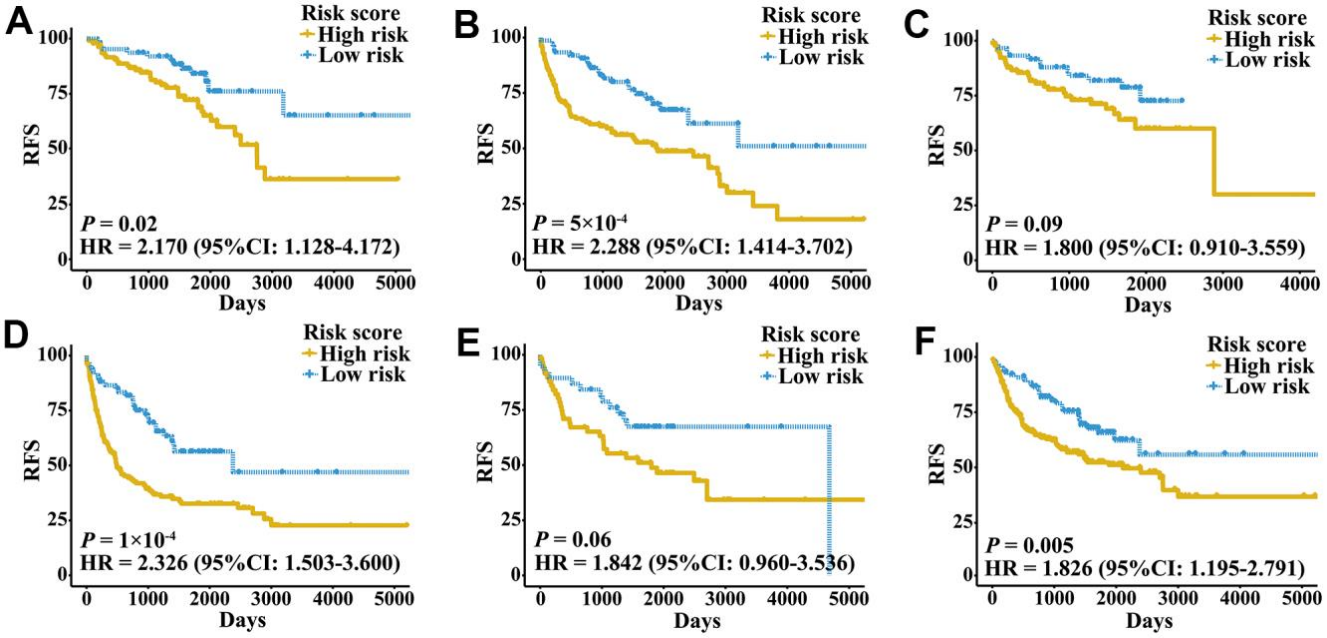
**Kaplan-Meier curves of patients with different clinical factors in the meta-validation cohort.** (**A**) RFS among patients with early-stage disease. (**B**) RFS among male patients. (**C**) RFS among patients younger than 60 years. (**D**) RFS among patients with late-stage disease. (**E**) RFS among female patients. (**F**) RFS among patients older than 60 years.

External validation cohorts were used to confirm the ability of the IRGPI to predict RFS of patients with three urologic cancers in different populations. The same IRGPs were used to calculate the IRGPI, and the patients were also classified into low- and high-risk groups. RFS of patients in low and high IRGPI groups were significantly different in the meta-validation cohort (Figure 3A, HR 3.326, 95% CI 2.623-4.217; *P* < 2×10^-16^), validation dataset of bladder cancer (Figure 3B, HR 3.987, 95% CI 2.641-6.019; *P* = 1×10^-12^), validation dataset of prostate cancer (Figure 3C, HR 3.277, 95% CI 2.275-4.719; *P* = 2×10^-11^), and validation dataset of kidney cancer (Figure 3D, HR 2.523, 95% CI 1.529-4.165; *P* = 2×10^-4^). Time-ROC curves showed stable predictive abilities, with 1-, 3-, 5-, and 10-year AUCs of 0.794, 0.764, 0.739, and 0.605, respectively (Supplementary Figure 2C). Similarly, the patients with high IRGPI and advanced tumor stage had the highest RFS among patients in the meta-validation cohort (Supplementary Figure 2D). Consistent with the training cohorts, the IRGPI was able to divide patients into significantly different groups in terms of RFS in mostly independent validation cohorts (Supplementary Figure 3). When considering patients with early- or late-stage disease, male patients and patients older than 60 years, the IRGPI remained highly prognostic, and a higher IRGPI score was associated with significantly worse prognosis (Figure 4). After adjusting for age and stage in Cox regression analyses, the IRGPI remained as an independent prognostic factor in the meta-validation cohort (HR 2.15, 95% CI 1.45-3.19; *P* = 0.000149; Supplementary Table 3).

### Functional analysis and infiltrating immune content related to the IRGPI

**Figure 5:**
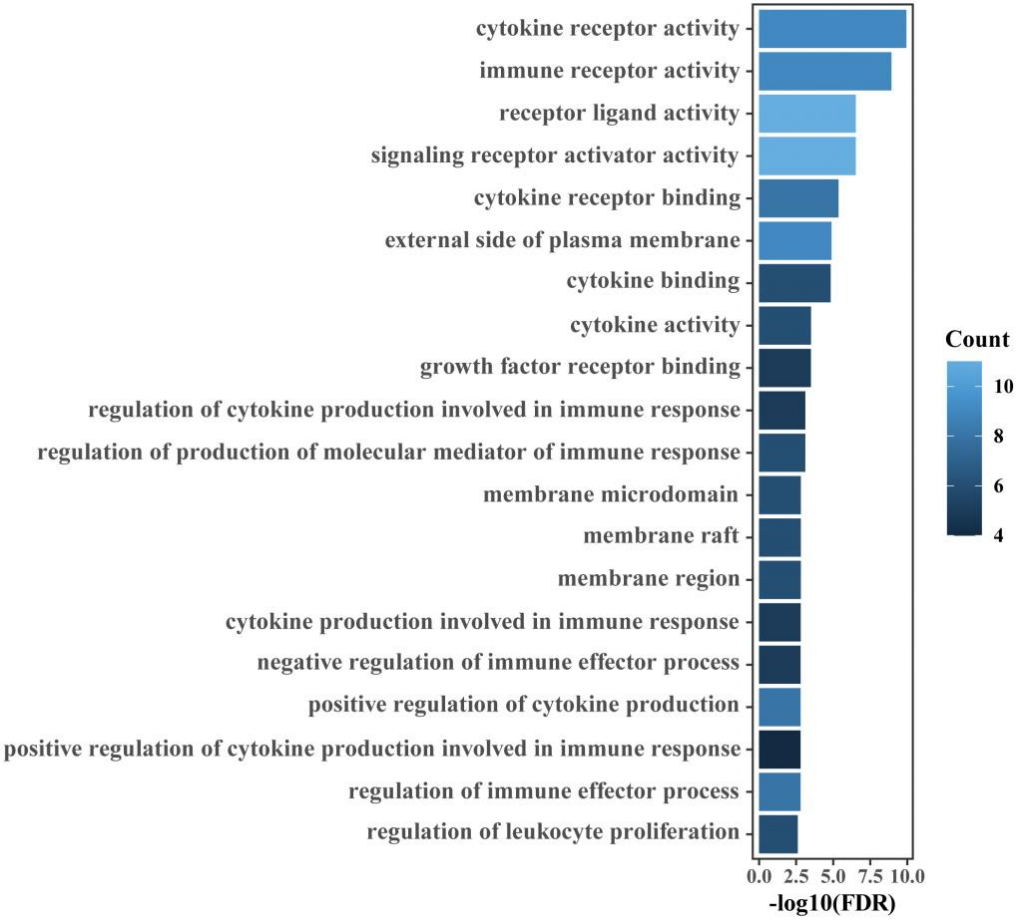
**GO enrichment analyses of IRGs relevant to the IRGPI.** The top 20 GO terms ranked by FDR are listed.

**Figure 6:**
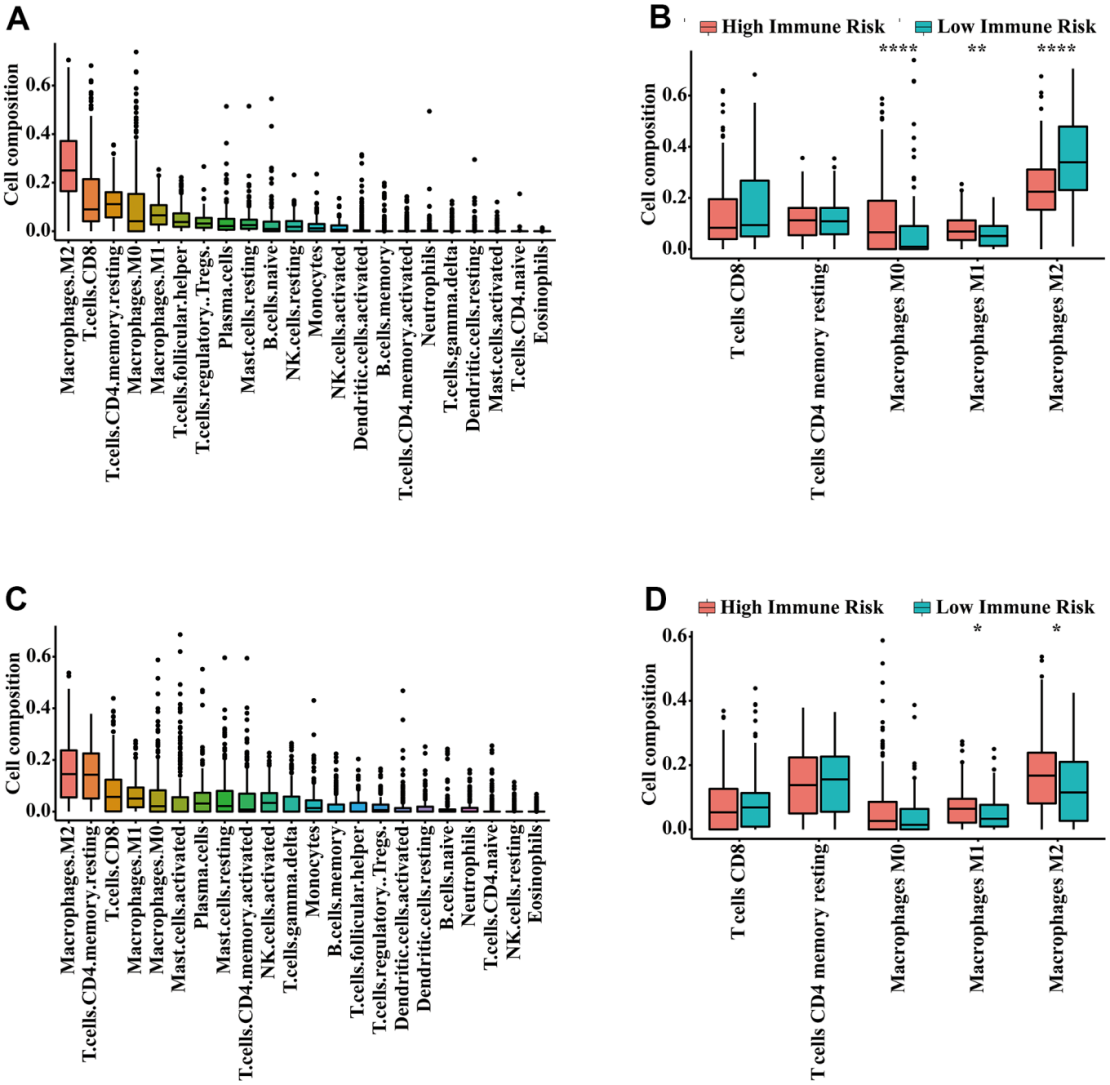
**Infiltrating immune content related to the IRGPI.** (**A**) The abundance of 22 immune cells in the meta-training cohort. (**B**) Immune cell abundance within each risk group in the meta-training cohort. The top five immune cells ranked by abundance are contrasted. (**C**) The abundance of 22 immune cells in the meta-validation cohort. (**D**) Immune cell abundance within each risk group in the meta-validation cohort. The top five immune cells ranked by abundance were contrasted. P-values were calculated with the Wilcoxon test (* *P* < 0.05, ** *P* < 0.01, *** *P* < 0.001).

The 41 unique IRGs relevant to the IRGPI in the meta-training cohort were mainly involved in the immune and inflammatory response, cytokine activity and chemotaxis (Supplementary Table 4 and Figure 5). Various immune infiltrates were enriched in the meta-training cohort, and Macrophages M2, T cells CD8, T cells CD4 memory resting, Macrophages M0 and Macrophages M1 showed higher abundance (Figure 6A). Among those, percentages of Macrophages M0, Macrophages M1 and macrophage M2 were significantly different between IRGPI risk groups (Figure 6B). Furthermore, those results were validated in the meta-validation cohorts in which the same five immune infiltrates with higher immune cell abundance were enriched (Figure 6C). The percentages of Macrophages M1 and macrophage M2 were also significantly different between IRGPI risk groups (Figure 6D).

### Nomogram based on the IRGPI and clinical factors

**Figure 7:**
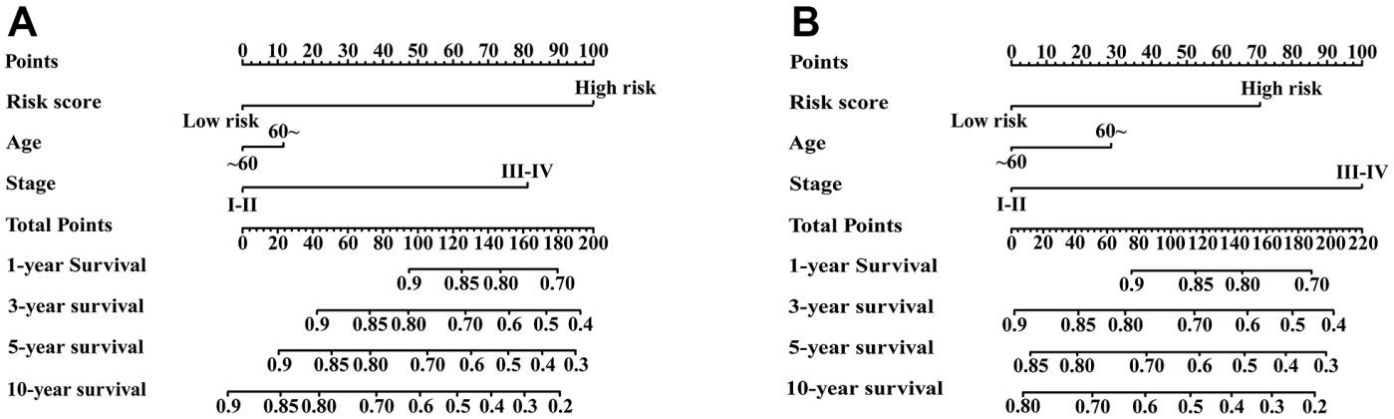
**Nomograms constructed for predicting 1-, 3-, 5-, and 10-year RFS.** (**A**) Nomograms predicting 1-, 3-, 5- and 10-year RFS rates among patients in the meta-training set. (**B**) Nomograms predicting 1-, 3-, 5- and 10-year RFS rates among patients in the meta-validation set.

Univariate Cox regression analysis showed that age, stage and IRGPI score were significant predictors of prognosis (Supplementary Table 3). To establish a quantitative approach for RFS prediction, we performed a nomogram based on prognostic factors (IRGPI, age, and stage) in the meta-training and meta-validation sets (Figure 7A, 7B). The reliability of the nomograms was evaluated by the calibration plot. As a result, the line-segments in the calibration plots were close to the 45° line, indicating an excellent agreement between the prediction and observation in the meta-training cohort (Supplementary Figure 4A–4D) and meta-validation cohort (Supplementary Figure 4E–4H).

## DISCUSSION

In this study, we developed a signature based on 25 IRGPs to simultaneously predict the prognosis of urinary cancer, including bladder, prostate, and kidney cancer. The signature showed a robust ability for predicting RFS of urinary cancers in training cohorts and multiple validation cohorts. Our signature could also distinguish different RFS in defined groups of patients (e.g., early-stage) in stratified analyses. The constructed nomogram based on the IRGPI score and clinical prognostic factors was able to quantitatively predict RFS rates of bladder, prostate and kidney cancer patients.

Patients with bladder [36], prostate [37] and kidney cancer [38] have substantial risk for relapse, even after surgical resection. The use of various adjuvant therapies, particularly in early-stage patients, remains disputable [39]. Reliable prognostic biomarkers are urgently needed to identify patients with a higher risk for relapse and select patients who have response to therapies. Many signatures based on gene expression were developed to predict the prognosis of bladder [40–42], prostate [43–45] and kidney cancer [46–48]. However, the common drawback in those studies were technical biases caused by the normalization of expression profiles derived from different platforms using RNA-Seq or microarrays. Based on the relative ranking of gene expression, the IRGPs signature in the present study focused on pairwise comparisons within the gene expression profile of samples. The need for data normalization was eliminated to the utmost extent, and technical biases between different platforms when combining multiple gene expression profiles were avoided. A few researchers have identified IRGPs signatures related to the prognosis of urinary cancer patients; for example, Fu et al. found a novel IRGPs signature that had significant prognostic value in predicting overall survival in bladder cancer [49]. Researchers in southern China have also developed an IRGPs to predict the prognosis of patients with papillary renal cell carcinoma [50]. However, these studies lacked sufficient validation due to the relatively small sample size, and could only predict the prognosis of single tumor. In this study, we integrated large-scale datasets from multiple platforms to identify the IRGPs signature. The signature could simultaneously predict RFS of three main urologic tumors, and was robust after being verified. Therefore, our signature can be expediently promoted to clinical usage.

The tumor immune microenvironment has been shown to be correlated with prognosis of bladder [22, 51], prostate [23, 52] and kidney cancer [24, 53]. In the era of immunotherapy, prognostic biomarkers relevant to the tumor immune microenvironment may break a new path for identifying novel prognostic biomarkers. In this study, most of the IRGs involved in our immune signature were cytokines, antimicrobials and cytokine receptors, which are closely related to immune response and inflammatory processes. Enrichment analysis also indicated that the IRGs relevant to the immune signature were mainly involved in the immune and inflammatory response and cytokine activity. An increased inflammatory microenvironment was found in the main tumors of the urinary system. This finding was consistent with previous studies showing that the formation and progression of tumors were related to an increased inflammatory microenvironment [54, 55]. Diverse immune cells such as neutrophils and macrophages are involved in the inflammatory response process of tumors. Macrophages have been shown to be correlated with poor prognosis in many cancers [56, 57]. In the present study, we found the infiltration level of macrophage M1 in the immune high-risk group significantly increased. It is possible that the dysregulated immune contexture may result in the survival differences observed between risk groups as defined by the IRGPI. Noteworthily, the infiltration level of M1 macrophages in the high-risk and low-risk group are inconsistent in researches [49, 50, 58–61]. Of these studies, two of them are consistent with our result [50, 58], three of them report that there is no significant difference [59–61], and only one research is contrary to ours [49]. The inconsistencies in the infiltration level of M1 macrophages may be related to study population differences, that is, the samples that generate risk scores are different.

To note, there were limitations in this study. Firstly, the 25 IRGPs prognostic signature was based on a retrospective study, although nine datasets were used for rigorous validation. Our results should be further validated in prospective cohorts with different sample attributes. Secondly, our prognostic signature also needs to be validated by quantitative real-time polymerase chain reaction before it can be applied clinically. Thirdly, we removed IRGPs with constant values to reduce the influence of batch effects between different platforms, but batch effects cannot be completely eliminated.

In conclusion, our 25 IRGPs signature is a promising and robust prognostic biomarker for predicting the RFS of bladder, prostate, and kidney cancer, including early-stage cancers. Moreover, this signature was associated with the infiltration of immune cell subsets and immune response, indicating the associations between the immune microenvironment and those cancers, and hence could help to formulate personalized immunotherapy strategy. Although the clinical utility of our signature needs to be validated in prospective studies, our study has nonetheless provided a panel of promising prognostic markers by integrating large-scale datasets.

## Supplementary Material

Supplementary Figures

Supplementary Tables 1-3

Supplementary Table 4
