# Supplementary material for: Identification and validation of an immune-related gene pairs signature for three urologic cancers
Source: Aging (Albany NY). 2022 Feb 10;14(3):1429–47. doi: 10.18632/aging.203886 (PMC8876921; doi:10.18632/aging.203886)
Supplement: Supplementary Figures [file aging-14-203886-s001.pdf]

SUPPLEMENTARY FIGURES

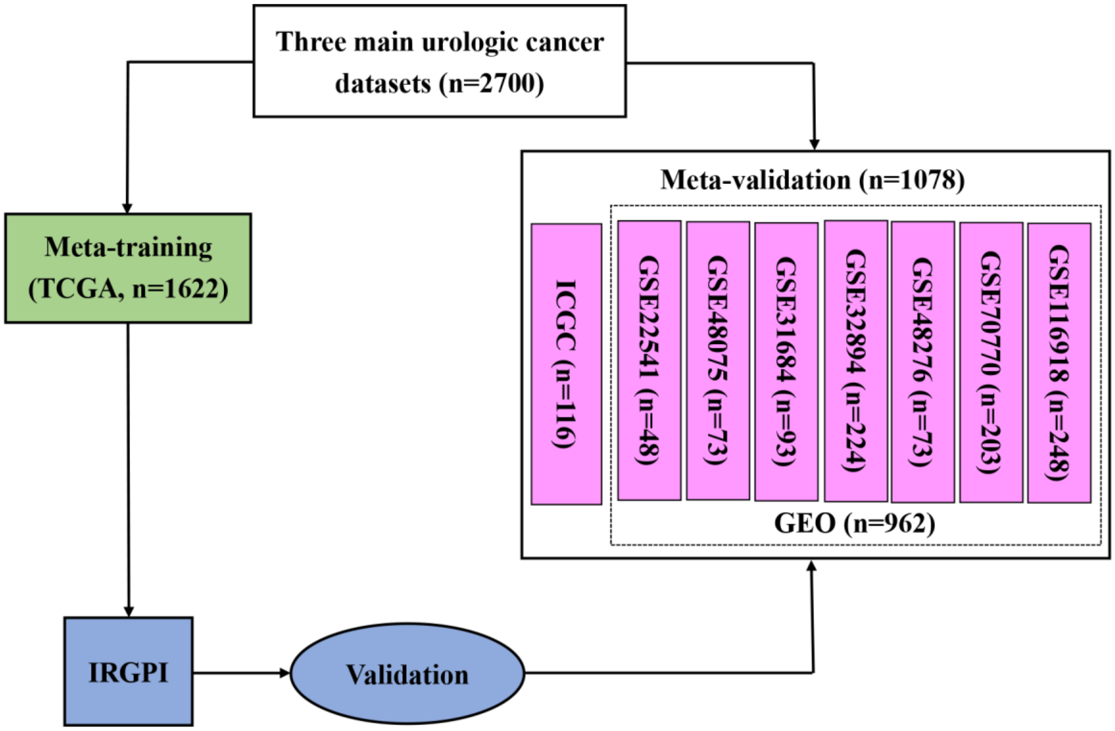

Supplementary Figure 1. Overview of the construction and validation of the immune-related gene pairs signature. Fourteen datasets were included in this study. Five TCGA datasets, including TCGA_BLCA (training cohort of bladder cancer), TCGA_PRAD (training cohort of prostate cancer), TCGA_KIRC, TCGA_KIRP and TCGA_KICH constituted the meta-training dataset. TCGA_KIRC, TCGA_KIRP and TCGA_KICH were combined for the kidney cancer cohort (training cohort of kidney cancer). Seven GEO datasets including GSE48075, GSE31684, GSE32894, GSE48276, GSE70770, GSE116918 and GSE22541, and two ICGC datasets, including ICGC_PRAD and ICGC_RECA, were merged to form a meta-validation dataset. GSE48075, GSE31684, GSE32894 and GSE48276 were combined in the validation dataset of bladder cancer (validation cohort of bladder cancer). GSE70770, GSE116918 and ICGC_PRAD were merged to form the validation dataset of prostate cancer (validation cohort of prostate cancer). GSE22541 and ICGC_RECA constituted the validation dataset of kidney cancer (validation cohort of kidney cancer). The meta-training dataset was used to build an immune-related gene pairs signature index (IRGPI). The IRGPI was verified on the meta-validation and independent validation datasets.

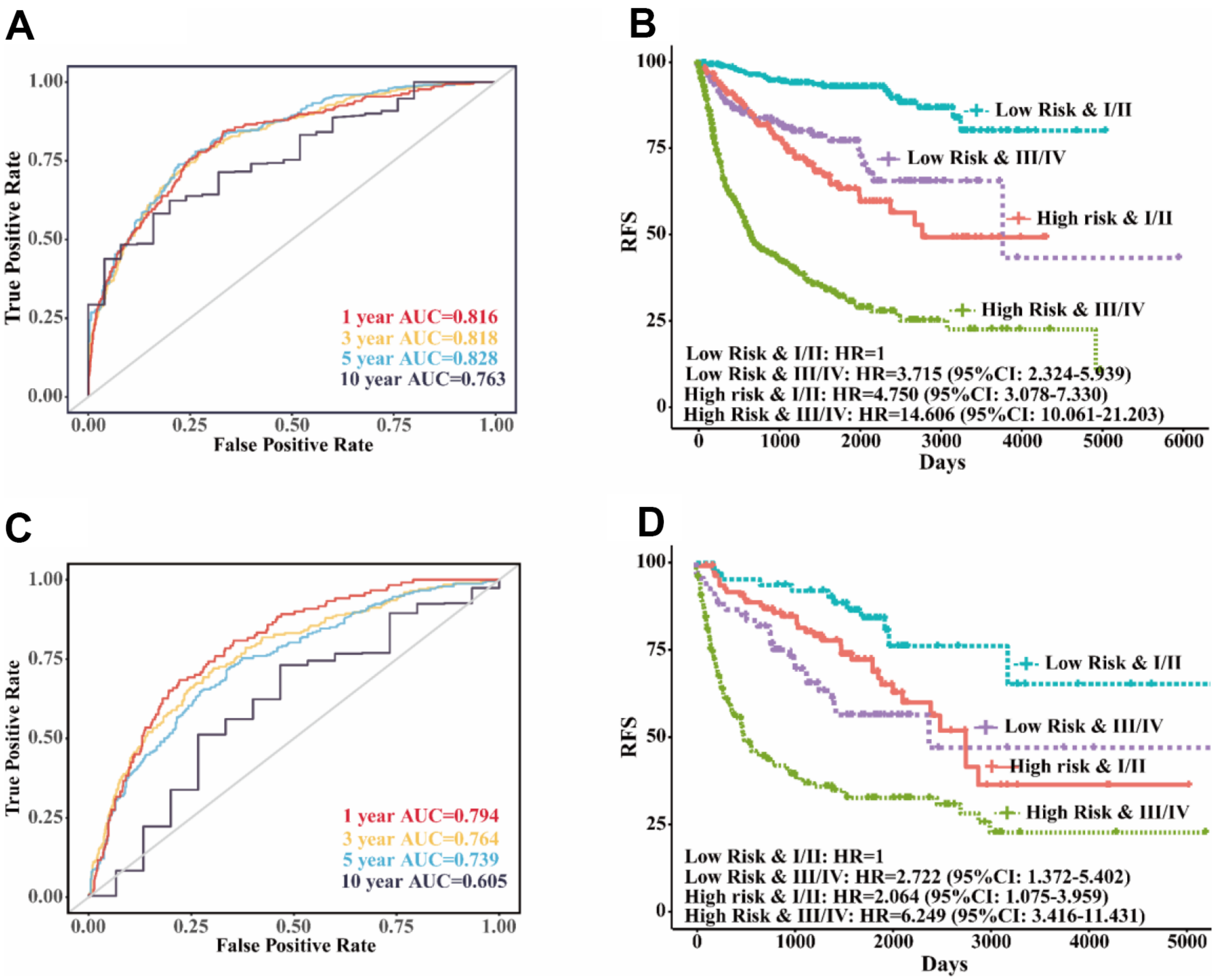

Supplementary Figure 2. Time-dependent ROC curves and Kaplan-Meier curves. (**A**) The time-dependent ROC curves of the IRGPI for 1-, 3-, 5- and 10-year RFS of patients in the meta-training cohort. (**B**) RFS among patients in the meta-training cohort stratified by IRGPI and tumor stage. (**C**) Time-dependent ROC curves of the IRGPI for 1-, 3-, 5- and 10-year RFS of patients in the meta-validation cohort. (**D**) RFS among patients in the meta-validation cohort stratified by IRGPI and tumor stage.

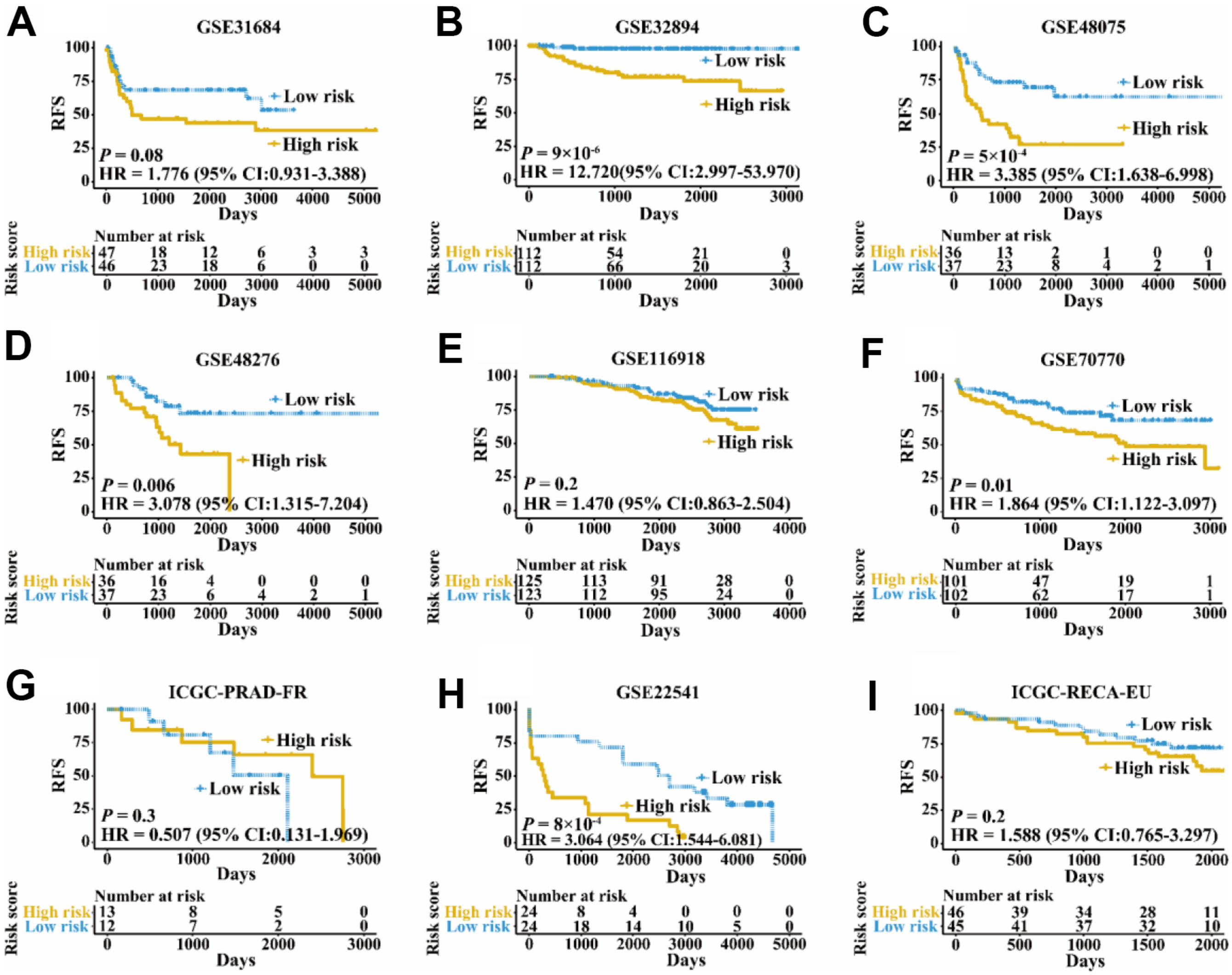

Supplementary Figure 3. Kaplan-Meier curves of patients in independent validation cohorts stratified by the IRGPI. (**A**) RFS among patients with bladder cancer in the GSE31684 cohort. (**B**) RFS among patients with bladder cancer in the GSE32894 cohort. (**C**) RFS among patients with bladder cancer in the GSE48075 cohort. (**D**) RFS among patients with bladder cancer in the GSE48276 cohort. (**E**) RFS among patients with prostate cancer in the GSE116918 cohort. (**F**) RFS among patients with prostate cancer in the GSE70770 cohort. (**G**) RFS among patients with prostate cancer in the ICGC-PRAD-FR cohort. (**H**) RFS among patients with kidney cancer in the GSE22541 cohort. (**I**) RFS among patients with kidney cancer in the ICGC-RECA-EU cohort. Hazard ratios (HRs) and 95% CIs were for high vs low immune risk. p-values comparing risk groups were calculated with the log-rank test.

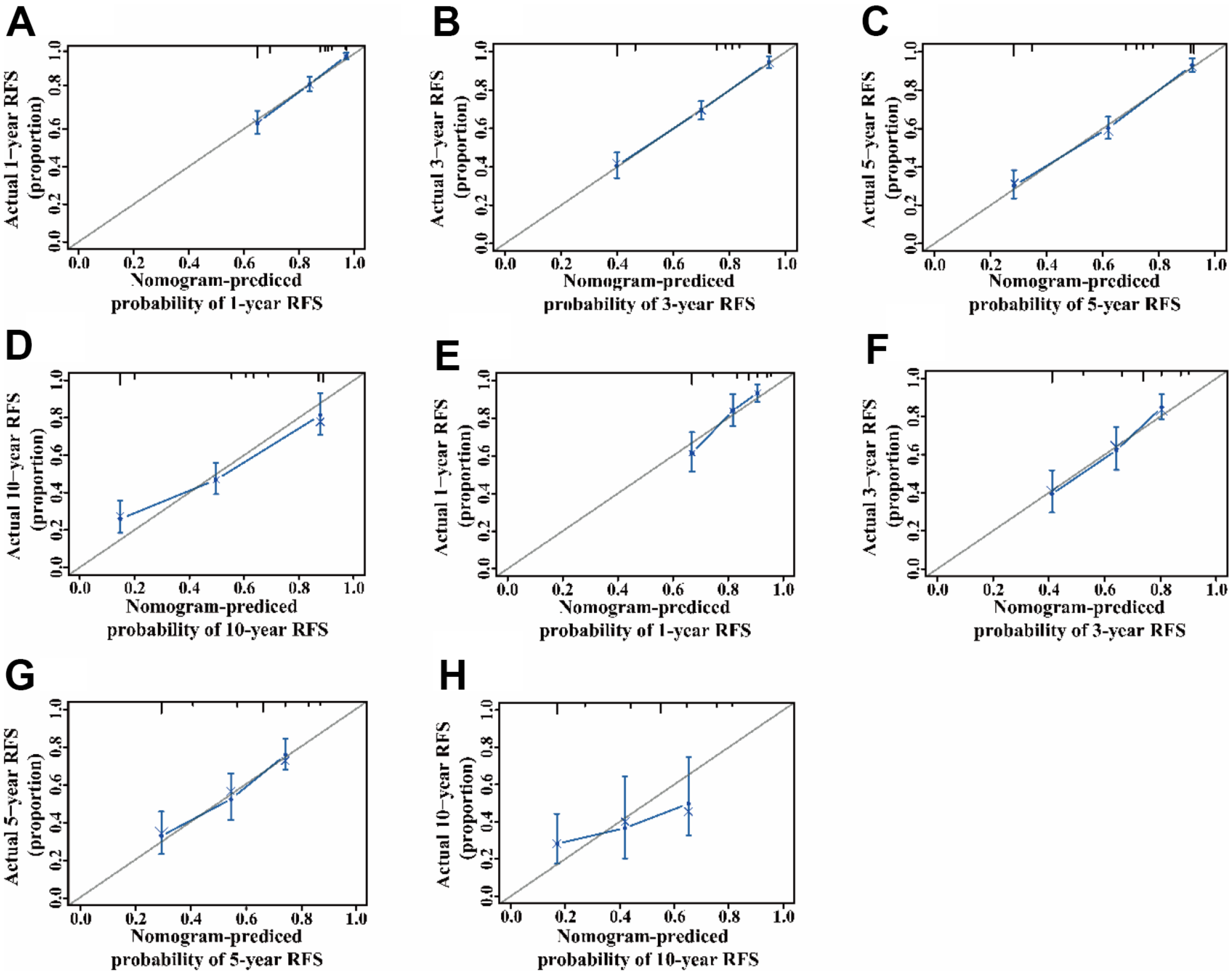

Supplementary Figure 4. Nomogram evaluation for predicting 1-, 3-, 5-, and 10-year RFS. (**A**–**D**) Calibration plots of the nomogram for predicting the probability of RFS at 1 (**A**), 3 (**B**), 5 (**C**) and 10 years (**D**) in the meta-training cohort. (**E**–**H**) Calibration plots of the nomogram for predicting the probability of RFS at 1 (**E**), 3 (**F**), 5 (**G**) and 10 years (**H**) in the meta-validation cohort.
