# Supplementary material for: Identification and validation of an immune-related gene pairs signature for three urologic cancers
Source: Aging (Albany NY). 2022 Feb 10;14(3):1429–47. doi: 10.18632/aging.203886 (PMC8876921; doi:10.18632/aging.203886)
Supplement: Supplementary Tables 1-3 [file aging-14-203886-s002.pdf]

SUPPLEMENTARY TABLES

Supplementary Table 1. Details about the data sets used in this study.

Accession number	Platform	Samples
**Urologic cancer**		**2,700**
**Bladder Cancer**		**835**
TCGA	Illumina HiSeq	372
GSE48075	Illumina HumanHT-12 V3.0 expression beadchip	73
GSE31684	Affymetrix Human Genome U133 Plus 2.0 Array	93
GSE32894	Illumina HumanHT-12 V3.0 expression beadchip	224
GSE48276	Illumina HumanHT-12 WG-DASL V4.0 R2 expression beadchip	73
**Prostate Cancer**		**888**
TCGA	Illumina HiSeq	412
GSE70770	Illumina HumanHT-12 V4.0 expression beadchip	203
GSE116918	Almac Diagnostics Prostate Disease Specific Array (DSA)	248
ICGC-PRAD-FR	Illumina HiSeq	25
**Kidney cancer**		**977**
TCGA Kidney Clear Cell Carcinoma	Illumina HiSeq	503
TCGA Kidney Papillary Cell Carcinoma	Illumina HiSeq	276
TCGA Kidney Chromophobe Carcinoma	Illumina HiSeq	59
GSE22541	Affymetrix Human Genome U133 Plus 2.0 Array	48
ICGC-RECA-EU	Illumina HiSeq	91

Supplementary Table 2. Model information about the IRGPI.

IRG 1	Full name	Immune processes	IRG 2	Full name	Immune processes	Coefficient
FASLG	Fas ligand	Multiple	FGF5	fibroblast growth factor 5	Cytokines	0.611523169778837
JAK2	Janus kinase 2	Antimicrobials	IL15RA	interleukin 15 receptor subunit alpha	Multiple	0.333805374153765
IREB2	iron responsive element binding protein 2	Antimicrobials	NEDD4	NEDD4 E3 ubiquitin protein ligase	Antimicrobials	0.557427035341619
BST2	bone marrow stromal cell antigen 2	Antimicrobials	SLC40A1	solute carrier family 40 member 1	Antimicrobials	-0.343127675750891
BIRC5	baculoviral IAP repeat containing 5	Antimicrobials	IL11RA	interleukin 11 receptor subunit alpha	Multiple	0.120504722606519
IL5RA	interleukin 5 receptor subunit alpha	Multiple	PDIA2	protein disulfide isomerase family A member 2	Antigen_Processing_and_Presentation	0.342856072023345
RSAD2	radical S-adenosyl methionine domain containing 2	Antimicrobials	IL13RA2	interleukin 13 receptor subunit alpha 2	Multiple	0.678808880744686
BIRC5	baculoviral IAP repeat containing 5	Antimicrobials	NR4A3	nuclear receptor subfamily 4 group A member 3	Cytokine_Receptors	-0.291520461858733
CXCL5	C-X-C motif chemokine ligand 5	Multiple	PLAU	plasminogen activator, urokinase	Multiple	-0.806211590266488
PDGFRA	platelet derived growth factor receptor alpha	Multiple	GNAI1	G protein subunit alpha i1	Antimicrobials	-0.279543844347446
IL21R	interleukin 21 receptor	Multiple	KLRD1	killer cell lectin like receptor D1	Multiple	-0.280214943593435
FABP3	fatty acid binding protein 3	Antimicrobials	LTB4R	leukotriene B4 receptor	Multiple	0.194642442980463
IFIH1	interferon induced with helicase C domain 1	Antimicrobials	IL1RAP	interleukin 1 receptor accessory protein	Multiple	0.185869442713303
BIRC5	baculoviral IAP repeat containing 5	Antimicrobials	MAP3K8	mitogen-activated protein kinase kinase kinase 8	TCRsignaling Pathway	-0.129389559702631

BIRC5	baculoviral IAP repeat containing 5	Antimicrobials	SEMA3F	semaphorin 3F	Multiple	-0.072138438403510
IL1RAP	interleukin 1 receptor accessory protein	Multiple	NR3C2	nuclear receptor subfamily 3 group C member 2	Cytokine_ Receptors	-0.0912124340222771
SEMA7A	semaphorin 7A (John Milton Hagen blood group)	Multiple	SECTM1	secreted and transmembrane 1	Cytokines	-0.343147031348337
IL20RB	interleukin 20 receptor subunit beta	Multiple	S100A8	S100 calcium binding protein A8	Antimicrobials	-0.396800456172257
LIFR	LIF receptor subunit alpha	Cytokine_Receptors	JAG1	jagged canonical Notch ligand 1	Cytokines	0.645240142760529
BIRC5	baculoviral IAP repeat containing 5	Antimicrobials	MICB	MHC class I polypeptide-related sequence B	Multiple	-0.071431251596447
BIRC5	baculoviral IAP repeat containing 5	Antimicrobials	TNFRSF10A	TNF receptor superfamily member 10a	Multiple	-0.0734371636311053
CCL22	C-C motif chemokine ligand 22	Multiple	GREM1	gremlin 1, DAN family BMP antagonist	Cytokines	0.279290394701392
FGF12	fibroblast growth factor 12	Cytokines	TNFSF4	TNF superfamily member 4	Multiple	0.379557014519608
IL13RA2	interleukin 13 receptor subunit alpha 2	Multiple	MPL	MPL proto-oncogene, thrombopoietin receptor	Cytokine_Receptors	-0.17903152209123
PCSK1	proprotein convertase subtilisin/kexin type 1	Antimicrobials	NUDT6	nudix hydrolase 6	Cytokines	-0.0916418175539433

Supplementary Table 3. Univariate and multivariate analyses of prognostic factors in terms of RFS.

Datasets	Variable	Univariate analysis		Multivariate analysis*	
		HR (95% CI)	*P* Value	HR (95% CI)	*P* Value
Meta-training	Age	1.56(1.28-1.90)	1.35×10^{-5}	1.18(0.96-1.45)	0.11
	Gender	1.07(0.86-1.34)	0.536	-	-
	Stage	5.05(4.00-6.38)	$< 2\times10^{-16}$	3.22(2.52-4.11)	$< 2\times10^{-16}$
	Immune risk	6.08(4.75-7.77)	$< 2\times10^{-16}$	4.22(3.23-5.52)	$< 2\times10^{-16}$
Meta-validation	Age	1.50(1.05-2.15)	0.0279	1.36(0.91-2.03)	0.135771
	Gender	0.99(0.69-1.42)	0.948	-	-
	Stage	2.96(2.12-4.14)	2.01×10^{-10}	2.94(2.02-4.29)	1.82×10^{-8}
	Immune risk	2.12(1.55-2.91)	2.57×10^{-6}	2.15(1.45-3.19)	0.000149

*Age, stage and immune risk were adjusted in multivariate analysis.
