# Supplementary material for: Identification and validation of an immune-related gene pairs signature for three urologic cancers
Source: Aging (Albany NY). 2022 Feb 10;14(3):1429–47. doi: 10.18632/aging.203886 (PMC8876921; doi:10.18632/aging.203886)
Supplement: Supplementary Table 4 [file aging-14-203886-s003.docx]

**Supplementary Table 4. The significant biological processes enriched by genes consisted in the IRGPI.**

| GO ID | Category | Description | FDR | Count |
| --- | --- | --- | --- | --- |
| GO:0004896 | MF | cytokine receptor activity | 1.15E-10 | 9 |
| GO:0140375 | MF | immune receptor activity | 1.18E-09 | 9 |
| GO:0048018 | MF | receptor ligand activity | 3.00E-07 | 11 |
| GO:0030546 | MF | signaling receptor activator activity | 3.00E-07 | 11 |
| GO:0005126 | MF | cytokine receptor binding | 4.37E-06 | 8 |
| GO:0009897 | CC | external side of plasma membrane | 1.31E-05 | 9 |
| GO:0019955 | MF | cytokine binding | 1.47E-05 | 6 |
| GO:0005125 | MF | cytokine activity | 0.000312241 | 6 |
| GO:0070851 | MF | growth factor receptor binding | 0.000319705 | 5 |
| GO:0002700 | BP | regulation of production of molecular mediator of immune response | 0.000763162 | 6 |
| GO:0002718 | BP | regulation of cytokine production involved in immune response | 0.000763162 | 5 |
| GO:0045121 | CC | membrane raft | 0.001528935 | 6 |
| GO:0098857 | CC | membrane microdomain | 0.001528935 | 6 |
| GO:0098589 | CC | membrane region | 0.001528935 | 6 |
| GO:0002367 | BP | cytokine production involved in immune response | 0.001558501 | 5 |
| GO:0002697 | BP | regulation of immune effector process | 0.001584042 | 8 |
| GO:0001819 | BP | positive regulation of cytokine production | 0.001584042 | 8 |
| GO:0002698 | BP | negative regulation of immune effector process | 0.001584042 | 5 |
| GO:0002720 | BP | positive regulation of cytokine production involved in immune response | 0.001584042 | 4 |
| GO:0070663 | BP | regulation of leukocyte proliferation | 0.002437126 | 6 |
| GO:0032102 | BP | negative regulation of response to external stimulus | 0.00578585 | 7 |
| GO:0006919 | BP | activation of cysteine-type endopeptidase activity involved in apoptotic process | 0.00578585 | 4 |
| GO:0002440 | BP | production of molecular mediator of immune response | 0.006947113 | 6 |
| GO:0070661 | BP | leukocyte proliferation | 0.007220701 | 6 |
| GO:0002702 | BP | positive regulation of production of molecular mediator of immune response | 0.007872488 | 4 |
| GO:0002456 | BP | T cell mediated immunity | 0.008518522 | 4 |
| GO:0001664 | MF | G protein-coupled receptor binding | 0.008583956 | 5 |
| GO:0036041 | MF | long-chain fatty acid binding | 0.008583956 | 2 |
| GO:0009615 | BP | response to virus | 0.009293782 | 6 |
| GO:0002703 | BP | regulation of leukocyte mediated immunity | 0.009293782 | 5 |
| GO:0043281 | BP | regulation of cysteine-type endopeptidase activity involved in apoptotic process | 0.009293782 | 5 |
| GO:0002699 | BP | positive regulation of immune effector process | 0.010266836 | 5 |
| GO:0002449 | BP | lymphocyte mediated immunity | 0.010786999 | 6 |
| GO:2000116 | BP | regulation of cysteine-type endopeptidase activity | 0.012776456 | 5 |
| GO:0043280 | BP | positive regulation of cysteine-type endopeptidase activity involved in apoptotic process | 0.014443469 | 4 |
| GO:0002704 | BP | negative regulation of leukocyte mediated immunity | 0.014443469 | 3 |
| GO:0048872 | BP | homeostasis of number of cells | 0.015234735 | 5 |
| GO:0043401 | BP | steroid hormone mediated signaling pathway | 0.015234735 | 4 |
| GO:0048863 | BP | stem cell differentiation | 0.015868985 | 5 |
| GO:0030522 | BP | intracellular receptor signaling pathway | 0.015868985 | 5 |
| GO:0052548 | BP | regulation of endopeptidase activity | 0.016594181 | 6 |
| GO:0070665 | BP | positive regulation of leukocyte proliferation | 0.016594181 | 4 |
| GO:2001056 | BP | positive regulation of cysteine-type endopeptidase activity | 0.016594181 | 4 |
| GO:0048008 | BP | platelet-derived growth factor receptor signaling pathway | 0.016594181 | 3 |
| GO:0032736 | BP | positive regulation of interleukin-13 production | 0.016594181 | 2 |
| GO:0022407 | BP | regulation of cell-cell adhesion | 0.017089014 | 6 |
| GO:0060485 | BP | mesenchyme development | 0.017623658 | 5 |
| GO:0050777 | BP | negative regulation of immune response | 0.017623658 | 4 |
| GO:0050922 | BP | negative regulation of chemotaxis | 0.017623658 | 3 |
| GO:0070486 | BP | leukocyte aggregation | 0.017623658 | 2 |
| GO:0031958 | BP | corticosteroid receptor signaling pathway | 0.017623658 | 2 |
| GO:0030215 | MF | semaphorin receptor binding | 0.018591349 | 2 |
| GO:0002683 | BP | negative regulation of immune system process | 0.019093784 | 6 |
| GO:0052547 | BP | regulation of peptidase activity | 0.019250786 | 6 |
| GO:0060326 | BP | cell chemotaxis | 0.020441996 | 5 |
| GO:0002709 | BP | regulation of T cell mediated immunity | 0.020441996 | 3 |
| GO:0002830 | BP | positive regulation of type 2 immune response | 0.020441996 | 2 |
| GO:0042110 | BP | T cell activation | 0.021091196 | 6 |
| GO:0003707 | MF | steroid hormone receptor activity | 0.021799343 | 2 |
| GO:0010950 | BP | positive regulation of endopeptidase activity | 0.022600811 | 4 |
| GO:0045499 | MF | chemorepellent activity | 0.023337929 | 2 |
| GO:0032496 | BP | response to lipopolysaccharide | 0.023402367 | 5 |
| GO:0030308 | BP | negative regulation of cell growth | 0.023402367 | 4 |
| GO:0022408 | BP | negative regulation of cell-cell adhesion | 0.023402367 | 4 |
| GO:0014032 | BP | neural crest cell development | 0.023402367 | 3 |
| GO:1901224 | BP | positive regulation of NIK/NF-kappaB signaling | 0.023402367 | 3 |
| GO:0061081 | BP | positive regulation of myeloid leukocyte cytokine production involved in immune response | 0.023402367 | 2 |
| GO:0032656 | BP | regulation of interleukin-13 production | 0.023402367 | 2 |
| GO:0032674 | BP | regulation of interleukin-5 production | 0.023402367 | 2 |
| GO:0034341 | BP | response to interferon-gamma | 0.023474752 | 4 |
| GO:0009755 | BP | hormone-mediated signaling pathway | 0.023474752 | 4 |
| GO:0010952 | BP | positive regulation of peptidase activity | 0.023474752 | 4 |
| GO:0014031 | BP | mesenchymal cell development | 0.023474752 | 3 |
| GO:0048864 | BP | stem cell development | 0.023474752 | 3 |
| GO:0014068 | BP | positive regulation of phosphatidylinositol 3-kinase signaling | 0.023474752 | 3 |
| GO:0070098 | BP | chemokine-mediated signaling pathway | 0.023474752 | 3 |
| GO:0014033 | BP | neural crest cell differentiation | 0.023474752 | 3 |
| GO:0002726 | BP | positive regulation of T cell cytokine production | 0.023474752 | 2 |
| GO:0032634 | BP | interleukin-5 production | 0.023474752 | 2 |
| GO:0032616 | BP | interleukin-13 production | 0.023474752 | 2 |
| GO:0023019 | BP | signal transduction involved in regulation of gene expression | 0.023474752 | 2 |
| GO:0002237 | BP | response to molecule of bacterial origin | 0.024267057 | 5 |
| GO:0071383 | BP | cellular response to steroid hormone stimulus | 0.02441087 | 4 |
| GO:0050866 | BP | negative regulation of cell activation | 0.024482064 | 4 |
| GO:0007159 | BP | leukocyte cell-cell adhesion | 0.02561098 | 5 |
| GO:0005164 | MF | tumor necrosis factor receptor binding | 0.026545952 | 2 |
| GO:0002460 | BP | adaptive immune response based on somatic recombination of immune receptors built from immunoglobulin superfamily domains | 0.026752143 | 5 |
| GO:0010810 | BP | regulation of cell-substrate adhesion | 0.026752143 | 4 |
| GO:1990868 | BP | response to chemokine | 0.026752143 | 3 |
| GO:1990869 | BP | cellular response to chemokine | 0.026752143 | 3 |
| GO:0032753 | BP | positive regulation of interleukin-4 production | 0.026752143 | 2 |
| GO:0097529 | BP | myeloid leukocyte migration | 0.028701461 | 4 |
| GO:0030593 | BP | neutrophil chemotaxis | 0.029751957 | 3 |
| GO:0048843 | BP | negative regulation of axon extension involved in axon guidance | 0.029751957 | 2 |
| GO:0060343 | BP | trabecula formation | 0.029751957 | 2 |
| GO:0050920 | BP | regulation of chemotaxis | 0.030133752 | 4 |
| GO:0048762 | BP | mesenchymal cell differentiation | 0.030133752 | 4 |
| GO:0030595 | BP | leukocyte chemotaxis | 0.031211483 | 4 |
| GO:0040013 | BP | negative regulation of locomotion | 0.031558656 | 5 |
| GO:0051271 | BP | negative regulation of cellular component movement | 0.032011417 | 5 |
| GO:1902668 | BP | negative regulation of axon guidance | 0.032011417 | 2 |
| GO:0002526 | BP | acute inflammatory response | 0.033797945 | 3 |
| GO:0050727 | BP | regulation of inflammatory response | 0.035816508 | 5 |
| GO:0001558 | BP | regulation of cell growth | 0.035816508 | 5 |
| GO:0050867 | BP | positive regulation of cell activation | 0.035816508 | 5 |
| GO:0045926 | BP | negative regulation of growth | 0.035816508 | 4 |
| GO:0030518 | BP | intracellular steroid hormone receptor signaling pathway | 0.035816508 | 3 |
| GO:0046916 | BP | cellular transition metal ion homeostasis | 0.035816508 | 3 |
| GO:1901222 | BP | regulation of NIK/NF-kappaB signaling | 0.035816508 | 3 |
| GO:0061082 | BP | myeloid leukocyte cytokine production | 0.035816508 | 2 |
| GO:0002828 | BP | regulation of type 2 immune response | 0.035816508 | 2 |
| GO:0002724 | BP | regulation of T cell cytokine production | 0.035816508 | 2 |
| GO:0032673 | BP | regulation of interleukin-4 production | 0.035816508 | 2 |
| GO:0046320 | BP | regulation of fatty acid oxidation | 0.035816508 | 2 |
| GO:0048841 | BP | regulation of axon extension involved in axon guidance | 0.035816508 | 2 |
| GO:0019838 | MF | growth factor binding | 0.036207807 | 3 |
| GO:0005504 | MF | fatty acid binding | 0.036207807 | 2 |
| GO:0017080 | MF | sodium channel regulator activity | 0.036207807 | 2 |
| GO:0030099 | BP | myeloid cell differentiation | 0.037137577 | 5 |
| GO:0051607 | BP | defense response to virus | 0.037137577 | 4 |
| GO:1990266 | BP | neutrophil migration | 0.037168982 | 3 |
| GO:0051539 | MF | 4 iron, 4 sulfur cluster binding | 0.037641088 | 2 |
| GO:0001952 | BP | regulation of cell-matrix adhesion | 0.03942886 | 3 |
| GO:0071621 | BP | granulocyte chemotaxis | 0.040054039 | 3 |
| GO:0014066 | BP | regulation of phosphatidylinositol 3-kinase signaling | 0.040054039 | 3 |
| GO:0032633 | BP | interleukin-4 production | 0.040054039 | 2 |
| GO:0051091 | BP | positive regulation of DNA-binding transcription factor activity | 0.041262781 | 4 |
| GO:0002701 | BP | negative regulation of production of molecular mediator of immune response | 0.041319291 | 2 |
| GO:0051090 | BP | regulation of DNA-binding transcription factor activity | 0.04242982 | 5 |
| GO:0048846 | BP | axon extension involved in axon guidance | 0.04242982 | 2 |
| GO:1902284 | BP | neuron projection extension involved in neuron projection guidance | 0.04242982 | 2 |
| GO:0007498 | BP | mesoderm development | 0.043517409 | 3 |
| GO:0032733 | BP | positive regulation of interleukin-10 production | 0.043517409 | 2 |
| GO:0032689 | BP | negative regulation of interferon-gamma production | 0.043517409 | 2 |
| GO:0008083 | MF | growth factor activity | 0.044732923 | 3 |
| GO:0032813 | MF | tumor necrosis factor receptor superfamily binding | 0.044732923 | 2 |
| GO:0008009 | MF | chemokine activity | 0.044732923 | 2 |
| GO:0004879 | MF | nuclear receptor activity | 0.044732923 | 2 |
| GO:0098531 | MF | ligand-activated transcription factor activity | 0.044732923 | 2 |
| GO:0042092 | BP | type 2 immune response | 0.045401415 | 2 |
| GO:0055076 | BP | transition metal ion homeostasis | 0.046202277 | 3 |
| GO:0071526 | BP | semaphorin-plexin signaling pathway | 0.046491143 | 2 |
| GO:0072210 | BP | metanephric nephron development | 0.046491143 | 2 |
| GO:1902042 | BP | negative regulation of extrinsic apoptotic signaling pathway via death domain receptors | 0.048391118 | 2 |
| GO:0072006 | BP | nephron development | 0.049332797 | 3 |
| GO:0051051 | BP | negative regulation of transport | 0.049755763 | 5 |
| GO:1904037 | BP | positive regulation of epithelial cell apoptotic process | 0.049755763 | 2 |
